# Ten high-priority areas for health policy research in 2026-2027

**DOI:** 10.1093/haschl/qxag154

**Published:** 2026-07-06

**Authors:** Jose F Figueroa, Loren Adler, William B Feldman, Yevgeniy Feyman, Simon F Haeder, A Jay Holmgren, Benedic Ippolito, Deborah A Marshall, Paul R Shafer, Kathryn A Phillips

**Affiliations:** Department of Health Policy and Management, Harvard T.H. Chan School of Public Health, Boston, MA 02115, USA; USC-Brookings Schaeffer Initiative for Health Policy, Brookings Institution, Washington, DC 20036, USA; Division of Pulmonary, Critical Care, Sleep Medicine, Clinical Immunology, and Allergy, Department of Medicine, University of California, Los Angeles 90095, USA; National Pharmaceutical Council, Washington, DC 20006, USA; Division of Health Services Management and Policy, The Ohio State University, Columbus, OH 43210, USA; Department of Medicine, University of California SanFrancisco, San Francisco, CA 94143, USA; American Enterprise Institute, Washington, DC 20036, USA; Department of Community Health Sciences, Cumming School of Medicine, University of Calgary, Calgary, Canada T2N 4N1; Department of Health Law, Policy, and Management, School of Public Health, Boston University, Boston, MA 02215, USA; UCSF Center for Translational and Policy Research on Precision Medicine (TRANSPERS), Department of Clinical Pharmacy, Philip R. Lee Institute for Health Policy Studies, University of California, San Francisco 94143, USA

**Keywords:** health policy, health reform, markets, pharmaceutical policy, technological innovation, climate change, health care delivery, Medicare, Medicaid

## Abstract

Health policy is undergoing a period of rapid transformation, driven by major health reforms, changing market structures, pharmaceutical policy developments, technological innovation, and evolving models of care delivery. These changes have profound implications for health care access, affordability, quality, and health system sustainability. In this editorial, we identify 10 priority areas for health services and policy research that we believe will shape policy debates and evidence needs in the coming years. These priorities are organized across 5 domains: health care access, coverage, quality and delivery system reform; corporatization and financialization of health care systems; pharmaceutical policy and health services research; international comparisons and cross-national learning; and innovation, technological advancements, and resiliency of health systems. Specific areas we discuss include the effects of the H.R.1 on Medicaid coverage and economic security; the future of Medicare Advantage; the impact of immigration policies on access; the consequences of increasing corporatization of health care; reforms to pharmaceutical pricing; the role of international comparative research; and the implications of precision nutrition, climate-related disruptions, and artificial intelligence for health policy and health system performance. Across these topics, we emphasize the need for rigorous, policy-relevant research that moves beyond descriptive analyses to evaluate mechanisms, implementation, and real-world impacts.

## Introduction

Health policy is currently undergoing a period of rapid and consequential transformation across the globe. This shift is being driven by a convergence of forces, including major legislative reforms, rapid changes in public and private markets, increasing corporatization of care delivery, and national interventions in pharmaceutical markets, among others. At the same time, global interdependence, climate-related disruptions, and advances in artificial intelligence and precision medicine are redefining how care is being delivered and the health and well-being of people. These shifts are not occurring in isolation, as they are interacting in ways that have important implications for access, affordability, equity, and the long-term sustainability of our health care systems.

In this editorial, we outline 10 priority areas for health services and policy research selected by the Editorial team of *Health Affairs Scholar: Emerging and Global Health Policy (“Scholar”)* for the coming years, which were informed by emerging policy developments, gaps in the current evidence base, and areas of growing relevance to patients, policymakers, and health system leaders. For some of these research areas, we build on our previous 2023 publication on “Ten health policy challenges for the next 10 years.”^1^ Priorities are then organized across 5 themes spanning coverage and delivery reform, market structure, pharmaceutical policy, international comparisons, and emerging national and global challenges arising from technological innovations and climate change.

Together, these priorities reflect areas where timely evidence and high-quality research can meaningfully inform health policy and clinical leaders' decisions in the years ahead. In this context, *Scholar* seeks rigorous, policy-relevant research that generates actionable insight for patients, policymakers, clinicians, health system leaders, and other relevant stakeholders across these research areas. As an open-access journal under the aegis of *Health Affairs*, our partner journal, we are committed to broad dissemination of research and advancing the policy impact of scholarship across diverse audiences. We are particularly interested in work that moves beyond descriptive trends to examine mechanisms, implementation, and the real-world effects of policy design.

## Health care access, coverage, quality, and delivery reform

Major federal and state policy changes in the United States are reshaping coverage, benefits, health care delivery models, costs, and administrative burdens in a way that may result in unforeseen consequences for health care markets. Coverage policy is not merely about insurance; it affects labor markets, household income, hospital solvency, and intergenerational mobility. Importantly, it also impacts health care quality, utilization, and outcomes of populations.^2-4^ Below, we describe in further detail 3 key areas of research inquiry for the years ahead.

### How will the “One Big Beautiful Bill Act” reshape coverage, access, outcomes, and economic security?

H.R. 1, also known as the One Big Beautiful Bill Act (OBBBA), represents one of the most significant retrenchments of public benefits in decades, with projected reductions of more than $900 billion in Medicaid spending over 10 years.^5,6^ States may respond with changes in reimbursement and/or benefits given shifts in how they can finance their share of costs^7^; these responses, in turn, may lead to adverse impacts for patient access.^8,9^ While much attention has focused on expected enrollment losses,^10^ the OBBBA is likely to have far-reaching public health consequences.

Evidence published in *Scholar* demonstrates that Medicaid expansion increased income among newly eligible adults, highlighting its role in economic security and upward mobility.^11^ Coverage reductions may therefore spill over to financial well-being, labor supply, and local economies. Before the OBBBA was passed, the Trump administration also revoked Biden-era health-related social needs guidance that allowed states to support nutrition, housing, and other needs through Medicaid, which will constrain their ability to begin or renew waivers supporting health through these types of non-medical interventions.^12,13^

Another critical area for research concerns administrative burdens.^14^ Recent research in *Scholar* on patient administrative burden underscores how procedural complexity can function as a de facto eligibility restriction.^15^ Much of the projected insurance disenrollment arises not from ineligibility but from new work reporting requirements and more frequent eligibility redetermination. In fact, nationwide work reporting requirements beginning in 2027 are projected by the Congressional Budget Office to be the largest source of Medicaid savings.^16^ Therefore, moving forward, it will be important to track how administrative burdens affect ongoing coverage losses, enrollment trajectories, physical and mental health, medical debt, uncompensated care, and rural hospital viability. In addition, there may be important cross-program spillovers, including interactions with food and housing benefits (eg, Supplemental Nutrition Assistance Program and Temporary Assistance for Needy Families).

### Are we getting value from Medicare Advantage?

Medicare Advantage (MA) now enrolls more than half of Medicare beneficiaries and plays a central role in federal health spending. Evidence from the Medicare Payment Advisory Commission (MedPAC) suggests that federal spending is higher for beneficiaries enrolled in MA than it would be if they were covered under Traditional Medicare, driven in part by coding intensity and favorable beneficiary selection.^17-21^ This has sparked an active policy debate over MA payment policy and the right balance between fiscal sustainability and enrollee welfare. *Scholar* recently published several papers that have informed this debate, including studies documenting how coding practices affect federal spending^22,23^ and the extent to which MA spending translates into more generous coverage for enrollees.^24^

Simultaneously, the policy landscape is shifting in ways that may significantly impact the MA market. The Centers for Medicare & Medicaid Services (CMS) has phased in a revised MA risk-adjustment model intended to reduce payments tied to diagnoses that are coded much more intensively in MA than in Traditional Medicare.^17,25,26^ In addition, the Inflation Reduction Act introduced major reforms to Medicare pharmaceutical benefits that have increased the costs of covering prescription drugs.^27^ Together, these and related policy changes are likely to alter plan costs, benefit generosity, beneficiary enrollment patterns, and federal spending.

### How are federal and state immigration policies shaping health care access, quality, and outcomes?

Recently, as described in recently published *Scholar* articles, federal and state policy changes are reshaping health care access in substantial ways,^28,29^ particularly for undocumented immigrant populations.^30^ However, studying immigrant access to health care is challenging given numerous changes occurring at once, including broader federal enforcement actions, heterogenous state-level coverage expansions and retrenchments, and new eligibility requirements for state and federally sponsored care. At the same time, the effects of policy changes targeting immigrants may also exhibit profound spillover effects. Given the prevalence of mixed-status families, policy changes directed at undocumented immigrants may also affect US citizens and their communities through increased access burdens, coverage instability, and reduced utilization. These changes could also hurt the US health care workforce, which depends upon immigrants in numerous roles.^31^ Moreover, recent changes in policies in states like Texas and Florida are pulling medical providers into the enforcement of immigration policies with potentially wide-ranging consequences.^32,33^

In this context, it will be important to track the impact of recent policy changes on insurance coverage, safety-net utilization, maternal and child health, and community health consequences. Tracking fiscal and hospital-level consequences, including uncompensated care and local public health capacity as well as impact on the health care workforce, will be critical.

## Corporatization and financialization of health care systems

Ownership and control increasingly shape incentives in health care delivery. Private equity acquisitions, insurer-provider integration, health system employment, and specialty practice roll-ups are changing how care is organized, financed, and delivered. Policymakers in the United States are grappling with how these ownership models affect prices, quality, competition, clinical decision-making, and clinician autonomy. These dynamics are not unique to the United States, as many countries are confronting similar tensions related to consolidation, financialization, and commercialization of health care,^34^ creating important opportunities for cross-national learning and comparative policy research.

### What does the ongoing corporatization of the health care system mean for patients, providers, and prices?

The corporatization of health care extends beyond traditional consolidation. Changes in ownership and control can alter incentives related to pricing, coding, referral patterns, site of care, contracting strategy, staffing, and risk-bearing arrangements. *Scholar* has published emerging evidence on how ownership structures, including private equity investments, influence market behavior,^35-38^ but the rapid evolution of these models creates an urgent need for research that can inform competition policy, payment reform, and regulatory oversight.

Corporatization may exert its effects through multiple channels at once. Private equity roll-ups in physician specialties may change local market concentration and bargaining leverage, while insurer acquisitions of provider groups may affect coding intensity, referral patterns, network design, and the flow of spending within vertically integrated firms. Health system employment of physicians may similarly reshape site-of-care decisions, negotiated prices, and clinicians' working conditions. Moving forward, it will be important to understand how these ownership forms affect spending, prices, utilization, coding, quality, access, workforce conditions, patient experience, and patient outcomes. Such timely information can help policymakers understand which forms of corporatization create efficiencies, which raise antitrust or regulatory concerns, and which policy tools can best protect patients and promote competition.

## Pharmaceutical policy and health services research

Pharmaceutical markets are undergoing structural changes following the Inflation Reduction Act and other measures aimed at lowering pharmaceutical costs both at the federal and state levels.^27,39,40^ For example, recent evidence published in *Scholar* found that the Inflation Reduction Act may disproportionately disincentivize postapproval research in small molecule drugs.^41^ Below, we highlight 2 recent developments in pharmaceutical policy in need of timely evidence to guide ongoing policy reform considerations.

### What are the consequences of most-favored nation drug pricing policies for innovation, access, and costs?

The Inflation Reduction Act marked a historic shift toward expanding the federal government's role in prescription drug pricing.^27^ New Most-Favored Nation–style models proposed by the Centers for Medicare and Medicaid Innovation would introduce, for the first time, reference pricing approaches that link what Medicare pays for drugs to prices abroad.^42^ These policies aim to reduce spending but may alter incentives for innovation, launch strategies, and global pricing dynamics.

Recent research published by *Scholar* has examined the early impacts of the Inflation Reduction Act on clinical trial activity in oncology, suggesting a potential reduction in manufacturer-initiated studies of new indications following approval.^41^ However, it remains unclear how Most-Favored Nation policies may affect the pharmaceutical research and discovery pipelines, clinical trial geography, patient access to medications, and international prices. Because drug markets are globally interconnected, pricing reforms in the United States may reverberate through multinational investment decisions. Rigorous empirical work will be needed to assess both savings and unintended consequences.

### How is the 340B Drug Pricing Program and its impact on access and spending evolving?

Established in 1992 to support safety-net providers, the 340B program has expanded in recent years to now encompass more than 40% of acute care hospitals in the United States.^43,44^ This growth has sparked litigation by manufacturers against covered entities and bipartisan proposals for reform.^43^ Researchers in *Scholar* have addressed different aspects of the 340B program, which is currently featured as a collection on our website. For example, prior work has focused on evaluating the limited role of contract pharmacies in improving access for patients.^45^

To help inform ongoing debates, it is essential to provide new evidence on whether 340B continues to achieve its statutory goals, how hospitals use 340B-generated revenues, and how reforms might better allocate resources to the hospitals and clinics most in need. Important questions also remain about interactions between 340B participation, hospital consolidation, site-of-care shifts, and overall drug spending. State and federal lawmakers need rigorous evidence to inform carefully crafted policies that improve the program without compromising the care of underserved patient populations.

## International comparisons and cross-national learning

### How can cross-country comparisons inform health system design and health outcomes?

Cross-national comparisons provide a powerful opportunity to understand how different health system designs, financing arrangements, regulatory approaches, and delivery models influence health care access, utilization, and population health.^46-49^ They can also help identify drivers of regional variation within countries, as recently highlighted in *Scholar*,^50,51^ and provide important perspectives on how differences in resource allocation and the scope of health services may affect performance. Additionally, international comparisons offer unique insights into how policy choices shape health system performance, financing, immigration patterns,^52^ and access to health care services across diverse contexts. For example, recent evidence in *Scholar* across 41 countries in sub-Saharan Africa, Latin America and the Caribbean, and Asia-Pacific found substantial disruptions to HIV-related services following the freezing of US foreign assistance in January 2025.^53^ These comparisons can help policymakers distinguish challenges that are country-specific from those that reflect broader structural issues, generating evidence that may inform reforms in the United States and abroad.

Furthermore, in certain fields, such as infectious diseases and rare diseases, international collaboration, interdependencies, and coordination can be essential.^54-56^ In research on rare diseases, for example, multinational data sharing and coordinated policy frameworks are needed for generating evidence at scale.^57^

Despite their value, cross-country comparative health policy research remains underdeveloped.^58^ Expanding this work is increasingly important as nations confront shared challenges, including aging populations, climate change, technological advancements including artificial intelligence, rising health care costs, workforce shortages, and persistent inequities in health outcomes. Comparative analyses that move beyond descriptive benchmarking to identify the mechanisms through which policies affect outcomes can generate actionable evidence for policymakers. Greater investment in rigorous cross-national research will be essential to inform future health system reforms and improve population health worldwide.

## Innovation, technological advancements, and resiliency of health systems

Health systems are entering a period in which emerging forces are not only adding complexity but also fundamentally reshaping the boundaries of policy, clinical care, and health care delivery. Advances in precision medicine, accelerating climate-related disruptions, and the rapid integration of artificial intelligence are altering how care is delivered, financed, and evaluated. For example, in *Scholar*, researchers have written about the use of artificial intelligence to improve administrative processes in Medicaid^59^ and how US hospitals are integrating it into their processes.^60^

These developments challenge existing regulatory frameworks, payment models, and evidence standards, which were largely designed for more uniform, stable, and analog systems of care. At the same time, they introduce new opportunities to improve outcomes, target interventions more effectively, and increase system efficiency. Policymakers are therefore confronted with the challenge of enabling innovation while ensuring that these transformations do not exacerbate disparities, undermine accountability, or outpace the evidence base needed to guide decision-making. Below, we further describe 3 important areas of interest.

### What are the effects and policy implications of individualizing nutrition-focused interventions?

The rise of GLP-1 drugs for obesity, as well as other nutrition-focused interventions, highlights limitations of one-size-fits-all approaches as many people do not respond to such interventions.^61^ Precision nutrition is an approach to identify and target individualized dietary recommendations and therapeutic interventions. Although the National Institutes of Health is investing research dollars into their “Nutrition for Precision Health Initiative,”^62^ there have been few publications on the health policy implications of this initiative, including how to identify, target, and successfully implement nutrition-focused interventions to those who will most benefit. By integrating genetics, microbiome composition, metabolic profiles, and environmental factors to predict how a person will respond to specific foods and drug therapies for disease prevention and optimal health, precision nutrition promises targeted interventions but raises complex questions about reimbursement, equity, and evidence standards. High costs and differential responsiveness risk widening disparities if not carefully addressed.

A priority is policy-oriented research examining how to evaluate nutrition-focused interventions, design coverage policies, and align payment models with individualized care. Work that addresses equity implications and implementation challenges is especially important as these technologies diffuse into routine care.

### How should health systems and payment models respond to climate change-driven disruptions?

Climate change is increasingly affecting not only population health but also the stability and performance of health care delivery systems.^63^ Extreme heat, wildfires, floods, and severe storms can disrupt infrastructure, reduce access to essential services, and strain workforce capacity. These disruptions are not evenly distributed, disproportionately affecting older adults, low-income populations, and those with chronic conditions who rely most heavily on continuous care.^64,65^ Recent evidence published in *Scholar* demonstrates that climate-related shocks are associated with increases in hospitalizations and mortality, underscoring the system-level consequences of environmental change.^64^ Additionally, prior work has found that more socially vulnerable communities are less likely to receive insurance relief payments for personal property following extreme-weather–related events.^66^

Despite these risks, health care systems and payment models remain largely unprepared for climate-related disruptions. A priority is research that evaluates investments in system resilience, including infrastructure hardening, supply chain adaptation, and workforce planning. Equally important is research on how payment models and regulatory frameworks can incentivize preparedness rather than reactive response. Studies linking climate events to utilization, spending, and disparities will be essential to inform both federal and state policy. Additionally, research that helps us build a more climate-resilient health system is strongly encouraged.

### What are the effects of artificial intelligence on health care costs, outcomes, and equity, and what are the implications for health policy?

Artificial intelligence is rapidly transforming multiple domains of health care, from automating administrative operations to clinical decision support and diagnostic tools to claims adjudication and utilization management. For example, recent work in *Scholar* discussed how artificial intelligence will augment the “Generalist-Specialist” model, arguing that these technologies will augment the ability of generalists to function at a specialist-level knowledge in some clinical scenarios.^67^ While these technologies hold promise for improving efficiency and quality, their adoption has outpaced the development of governance frameworks, payment models, and evaluation standards.^68^ Key questions remain about how to assess the clinical value of artificial intelligence tools, how to monitor their performance and use long-term, how to integrate them into reimbursement systems, how patients employ these tools to manage their own care, and how to ensure accountability when decisions are partially or fully automated.

Availability of new technologies also raises important concerns related to equity, bias, and transparency. Algorithms trained on incomplete or biased data may reinforce existing disparities, while opaque decision-making processes can make oversight challenging.^69^ Therefore, it will be important for researchers to generate evidence on how artificial intelligence will affect the quality of care, clinician workload, costs of care, and patient outcomes.

## Conclusion

Taken together, these priority areas reflect both enduring challenges in health policy and emerging forces that are reshaping the future of health care delivery, financing, and population health. Across these domains, there remains a critical need for rigorous, policy-relevant evidence that not only documents trends but also explains mechanisms, evaluates real-world impacts, and informs actionable solutions. At *Scholar*, we welcome submissions in the coming years on these topics. While we highlight these areas as priorities for evidence generation, they are not exhaustive. *Scholar* will continue to welcome high-quality submissions across the full breadth of health policy research. As the policy landscape continues to evolve, advancing evidence across these areas will be essential to promoting access, improving quality and outcomes, ensuring affordability and equity, and strengthening the resilience of health systems.

## Supplementary Material

qxag154_Supplementary_Data
